# Supplementary material for: Musculoskeletal pain affects the age of retirement and the risk of work cessation among older people
Source: PLoS One. 2024 Mar 20;19(3):e0297155. doi: 10.1371/journal.pone.0297155 (PMC10954148; doi:10.1371/journal.pone.0297155)
Supplement: Attachment — Submitted filename: Response to Reviewers.docx [file pone.0297155.s002.docx]

Academic Editor: Please review your reference list to ensure that it is complete and correct. If you have cited papers that have been retracted, please include the rationale for doing so in the manuscript text, or remove these references and replace them with relevant current references. Any changes to the reference list should be mentioned in the rebuttal letter that accompanies your revised manuscript. If you need to cite a retracted article, indicate the article’s retracted status in the References list and also include a citation and full reference for the retraction notice.

**We have reviewed the reference list and made the following changes:**

Added Waddell & Burton 2006, Taylor 2017 due to omission from reference list.

Amended De Wind 2014 to De Wind 2013 in the manuscript (the reference list is correct to cite both).

Amended Bevan 2015 to Bevan 2015b, due to oversight.

Added Fimland et al. 2018 in introduction due to reviewer 3’s suggestion to focus more on musculoskeletal pain.

Reviewer #2: The comments raised in the previous version have been correctly addressed. This revised manuscript is easy to read and to understand.

**We thank reviewer 2 for the comments and suggestions, which we believe have greatly improved the paper.**

Reviewer #3: I had the opportunity to review this interesting manuscript on effects of MSK pain on retirement age among older people. The idea is interesting and the manuscript is well-written. There are a few areas where I believe some enhancements could further elevate the quality of the manuscript.

**Thank you for your comments and suggestions. We have revised the manuscript accordingly.**

1. According to the title the study aimed to evaluate the effects of MSK pains on work cessation and more focus should be place on MSK pains in the introduction section rather than discussing chronic pain in general.

**Thank you for bringing this up. We have made changes to the introduction to highlight the manuscript’s focus on MSK pain, see page 3.**

2. Please briefly describe the sampling and data collection process.

**We have added additional details pertaining to the sampling and data collection process on pages 5 and 6.**

3. Please consider showing the flow of participant in a figure (method section – participant subheading).

**Thank you for this suggestion. We have included a figure.**

4. Please move the variables subheading to the next line.

**We have moved the subheadings to the next line.**
